# Same-gender distractors are not so easy to reject: ERP evidence of gender categorization

**DOI:** 10.3758/s13415-018-0607-3

**Published:** 2018-05-07

**Authors:** Tamara Rakić, Melanie C. Steffens, Holger Wiese

**Affiliations:** 1 Psychology Department, Fylde College, Lancaster University, Lancaster, LA1 4YF UK; 2 University of Koblenz-Landau, Koblenz-Landau, Germany; 3 Durham University, Durham, UK

**Keywords:** Social categorization, Gender categorization, ERP, “Who-said-what” paradigm

## Abstract

Social categorization appears to be an automatic process that occurs during person perception. Understanding social categorization better is important because mere categorization can lead to stereotype activation and, in turn, to discrimination. In the present study we used a novel approach to examine event-related potentials (ERPs) of gender categorization in the “Who said what?” memory paradigm, thus allowing for a more in-depth understanding of the specific mechanisms underlying identity versus categorization processing. After observing video clips showing a “discussion” among female and male targets, participants were shown individual statements, each accompanied by one of the discussants’ faces. While we measured ERPs, participants had to decide whether or not a given statement had previously been made by the person with the accompanying face. In same-person trials, statements were paired with the correct person, whereas in the distractor trials, either a same-gender or a different-gender distractor was shown. As expected, participants were able to reject different-gender distractors faster than same-gender distractors, and they were more likely to falsely choose *yes* for a same-gender than for a different-gender distractor. Both findings indicate gender-based categorization. ERPs, analyzed in a 300- to 400-ms time window at occipito-temporal channels, indicated more negative amplitudes for *yes* responses both for the same person and for same-gender distractors, relative to different-gender distractors. Overall, these results show gender-based categorization even when the task was to assess the identifying information in a gender-neutral context. These findings are interpreted as showing that gender categorization occurs automatically during person perception, but later than race- or age-based categorization.

Social categorization into “us” and “them,” “men” and “women,” “Black” and “White,” “old” and “young” is the *conditio sine qua non* of unequal treatment of other people. Social-group stereotypes can only be applied after people have been categorized into the respective social groups. Social categorization often appears to be a fast and automatic process. To examine the automaticity of social categorization, researchers have developed sophisticated behavioral paradigms. In the “Who-said-what?” paradigm (WSW), after a discussion, memory errors are used to determine which social categories have been activated (Taylor, Fiske, Etcoff, & Ruderman, 1978). For example, if statements women originally made are falsely assigned to other women more than to men (and vice versa), it is concluded that gender categorization has occurred during the discussion. According to this reasoning, gender categorization during study should be visible also in differential neural responses during a memory test when same-gender versus different-gender distractors are presented. The aim of the present research was to test this hypothesis by measuring event-related potentials (ERPs). In the following paragraphs, we introduce social categorization, the WSW paradigm, and previous research using ERPs to study related questions.

Rosch’s (1973) pioneering work has made the human mind’s propensity to group together similar things and put them into categories a truism in psychology. Similarly, social categorization is humans’ tendency to sort other people into social categories. A single individual belongs to many social categories at once (e.g., being a woman, German, psychologist, and football fan). The “big three” of those social categories (i.e., gender, age, and ethnicity/race) appear to be rather chronically accessible (e.g., Fiske & Neuberg, 1990; but see Wiese, Schweinberger, & Neumann, 2008b), whereas other categories become salient depending on their situational accessibility (e.g., a singleton player of one football team among many who belong to a different team, or a conversation about football). Social categorization is often considered a double-edged sword. On the one hand, it appears inevitable, in order to simplify the world’s complexity. On the other hand, it may lead to stereotyping, biases in impression formation, false assumptions concerning the homogeneity of out-groups, and other cognitive biases that may ultimately lead to discrimination (for a review, see Steffens & Viladot, 2015). Several models specify how certain social categories are selected upon encountering individuals (e.g., Brewer, 1988; Fiske & Neuberg, 1990; Kunda & Thagard, 1996). Importantly, a widely shared assumption is that social categorization often is an automatic process that cannot be avoided.

How can the automaticity of social categorization be investigated? One of the methods most often used is the “Who said what” (WSW) paradigm (Taylor et al., 1978). During a discussion phase, participants are asked to form an impression of a group of individuals. These are presented one by one, each making several statements in a random order. Later, a surprise recognition test follows, and participants are asked to assign each statement to the person who made it. It is taken as evidence of gender categorization if statements that were made by women are more often falsely assigned to other women than to men, and statements made by men are more often falsely assigned to other men than to women. As another example, if statements that accented speakers made are falsely assigned to accented speakers more often than to those speaking standard language, accent-based categorization appears to have occurred (Rakić, Steffens, & Mummendey, 2011). Gender categorization is typically observed in the WSW paradigm (e.g., Klauer & Wegener, 1998), particularly if gender-related topics were discussed (e.g., Klauer, Ehrenberg, & Wegener, 2003). In the present study, we hypothesized that gender categorization during study should be manifest in different patterns of event-related brain potentials (ERPs) during recognition.

ERPs reflect transient voltage changes in the human electroencephalogram time-locked to a specific event, such as the presentation of a visual stimulus. The different components of ERPs reflect neural correlates of the various processing stages following stimulus presentation. They can therefore provide information about the specific mechanisms underlying an experimental effect. Recently, researchers have begun to study ERPs to find out more about the neural correlates of social perception and categorization (Bartholow, Pearson, Gratton, & Fabiani, 2003; Ito, Thompson, & Cacioppo, 2004; Ito & Urland, 2005; for a review, see Ito, 2011). For instance, it has been reported that the “race” of unambiguous faces is perceived faster than face gender (Ito & Urland, 2003; Willadsen-Jensen & Ito, 2006). Specifically, whereas the central N100 component was increased for Black relative to White faces, the subsequent P200 component was larger for Black than for White and for male than for female faces (Ito & Urland, 2003). In line with the latter finding, the occipito-temporal N170 (Bentin, Allison, Puce, Perez, & McCarthy, 1996), which presumably reflects the negative counterpart of the fronto-central P200 (Joyce & Rossion, 2005; Wiese, 2012), has been observed to be larger for other-race faces (e.g., Herrmann et al., 2007; Wiese, Kaufmann, & Schweinberger, 2014). The N170 is typically interpreted as reflecting early stages of face processing, prior to the identification of individual faces (e.g., Eimer, 2011). Accordingly, the N170 may reflect a neural correlate of categorizing face stimuli into ethnic groups. At the same time, reports of differential N170 amplitudes for male versus female faces are scarce (see Wolff, Kemter, Schweinberger, & Wiese, 2014), and the component has been observed to not reflect the discrimination of face gender (Mouchetant-Rostaing & Giard, 2003; Mouchetant-Rostaing, Giard, Bentin, Aguera, & Pernier, 2000). In contrast, others have reported that ERPs reflected ongoing gender categorization both at an early (N170) and at a later (P300) processing stage; this pattern was found when the task was related to explicit gender categorization or unrelated (i.e., dot detection; Tomelleri & Castelli, 2012).

Directly following the N170, a positive-going peak is usually observed at occipito-temporal channels, with a maximum at approximately 250 ms. This occipito-temporal P2 is typically interpreted as reflecting the processing of spatial information in faces (Mercure, Dick, & Johnson, 2008; Schweinberger & Neumann, 2016). It is larger for own-race faces in participants without particular expertise for other-race faces, but not in other-race face “experts” (Stahl, Wiese, & Schweinberger, 2008). Moreover, although a previous study observed this component to differentiate between later remembered and forgotten own-gender versus other-gender faces (Wolff et al., 2014), more recent studies have not found a correlate of gender processing in the occipito-temporal P2 (Wiese & Schweinberger, 2018).

The negative shift following the P2, the so-called N250 (approximately 250–400 ms), is often assumed to reflect the processing of faces’ individual identity. Thus, famous faces have been observed to elicit more negative amplitudes than unfamiliar faces (Andrews, Burton, Schweinberger, & Wiese, 2017; Gosling & Eimer, 2011) and preexperimentally unfamiliar faces elicit larger N250 amplitudes with increasing familiarity in the course of learning experiments (Kaufmann, Schweinberger, & Burton, 2009; Tanaka, Curran, Porterfield, & Collins, 2006). Interestingly, the N250 is sensitive to priming. For instance, N250 is more negative if a familiar face is preceded by the face of the same relative to a different person (Schweinberger, Huddy, & Burton, 2004). In case of the WSW paradigm, the presentation of a specific sentence during test may not prime the perceptual representation of the person who said this sentence, because the N250 priming effect (N250r) does not cross stimulus domains (e.g., from written words to faces). At the same time, one might assume that “cross-modal” effects are observed in the WSW paradigm, as only a very small set of face images is used, and participants could activate stored representations of these images when presented with identity-specific word material. If so, this should particularly be the case if the correct facial (and gender) identity is actually remembered. If a specific sentence is not associated with a specific face, the correct face will not be remembered and no preactivation should occur, resulting in less negative N250 amplitudes. Accordingly, the N250 is a potential candidate for reflecting the correct identification of individuals within this paradigm.

## The present research

The novelty of the present experiment is the combination of different experimental methods in gender-based categorization; namely, it tested whether the WSW paradigm goes along with observable changes in the N170, P2, and N250 time ranges. Briefly, during the study phase participants observed, on the computer screen, a discussion among male and female students. During the recognition phase, participants were presented one by one with sentences; each had been said by a specific discussant during the study phase. After each statement, a picture of one of the discussants was presented. Then participants decided within a time window whether or not this had been the person who made the statement. ERPs were recorded when the photo of the person who had said this sentence, or alternatively the photo of a different person, appeared. Accordingly, we analyzed responses to the same person (same identity, same gender), as well as to same-gender (different identity, same gender) and different-gender (different identity, different gender) distractors.

First, we expected that participants would fail to correctly respond within the 3,000-ms time window more often when the decision was more difficult. Therefore, same-gender distractors should yield more response omissions (Hypothesis 1) and slower responses (Hypothesis 2) as compared to different-gender distractors. Moreover, false positive responses should be more likely toward same-gender distractors than toward different-gender distractors, indicating social-category memory (Hypothesis 3). These first three hypotheses would replicate previous findings on gender-based categorization from memory research. With respect to ERPs, we assumed that when the correct person is remembered, the face representation of this individual person is preactivated when the sentence is presented at test. We therefore predicted for the same-person condition that the N250 for a correct *yes* response would be more negative than that to an incorrect *no* response. At the same time, the activation of a specific identity representation should be different from the activation of the broader gender category. Accordingly, the N250 was assumed to be more negative for correct same-person responses than for incorrect *yes* responses in the same-gender condition (in which the correct gender category was activated) (Hypothesis 4). Finally, following the logic of the WSW paradigm, categorization can be inferred when an error in the same-gender condition is different from an error in the different-gender condition (because in the first case the error is related to the activation of the correct gender category, and in the second it is not). At the same time, for gender categorization, ERPs to an error in the same-gender condition should look similar to those to a correct response in the same-person condition (as in both cases, we would assume that correct gender information was activated). We therefore expected ERPs to *yes* responses to differentiate between same-person/same-gender test faces on the one hand and different-gender test faces on the other hand. On the basis of the previous literature, we found it difficult to predict the exact ERP component in which this pattern should arise, but given that the majority of studies have not reported gender effects in the N170, we assumed that it would occur in a subsequent time window (Hypothesis 5). Taken together, this pattern would yield evidence that gender had been processed during study and remembered.

## Method

### Ethics statement

Ethical approval for this study was obtained from the Ethics Commission, University of Jena, Faculty of Social and Behavioral Sciences (number FSV 12/02). All participants gave their informed consent before starting the experiment.

### Participants and design

After excluding one participant who provided no responses within the time window of 3,000 ms, the remaining participants were 22 students at a large German university, among them 17 women (age range: 20–29 years, *M*_*age*_ = 24.00, *SD* = 2.76). They were invited to take part in an electroencephalography (EEG) study on person perception and received €10 as compensation.

The independent variables were the person depicted in the test phase (stimulus category: same person, same-gender distractor, different-gender distractor), yielding a three-level factorial design with repeated measures. Dependent variables were response omissions (due to time out), reaction times, responses (*yes* and *no*, to indicate if the displayed face is of the person that previously made a given statement), and ERPs in three time windows. Given the within-subjects design, the sample size was determined beforehand on the basis of the reasoning that a minimum of *N* = 20 would be needed to obtain reliable data (Simmons, Nelson, & Simonsohn, 2011). More precisely, given *α* = .05 and 22 participants, large main effects of *f* = 0.40 could be detected with a probability of 1 – *β* = .89 assuming a small correlation (*r* = .20) among the repeated measures (Cohen, 1977; Faul, Erdfelder, Lang, & Buchner, 2007).

### Materials

**Fig. 1:**
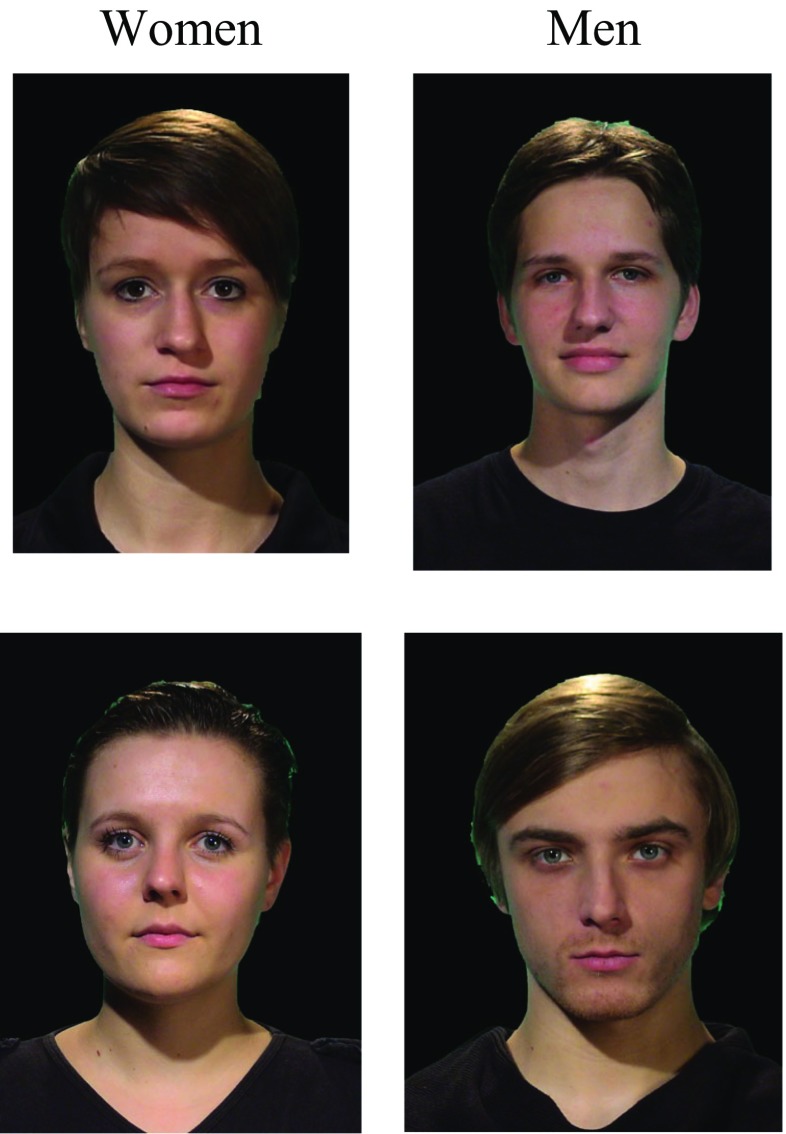
Examples of targets used in the experiment

We used video clips of eight target people (four women and four men; see Fig. 1 for example pictures). To make sure that the chosen targets were appropriate, we tested independently the perceived femininity or masculinity (depending on the target’s gender) of their appearance and voice on a 7-point Likert scale (1 = *not at all* to 7 = *very much*). The testing of these targets was completed in a bigger stimulus testing session in which different groups of participants rated different stimuli; this is reflected in the different degrees of freedom for the different tests. A total of *N* = 97 participants participated in the stimulus pretests (36 men, 61 women; age range: 18 to 34, *M*_*age*_ = 22.67, *SD* = 3.26).

On the basis of appearance and voice, there was no difference in perceived femininity (*M* = 5.29, *SD* = 1.32, and *M* = 5.63, *SD* = 1.25, respectively) or masculinity (*M* = 5.26, *SD* = 1.34, and *M* = 5.77, *SD* = 1.03, respectively), *t*(76) = – 0.04, *p* = .97, *d* = 0.009, and *t*(92) = – 1.15, *p* = .25, *d* = 0.24. Perceived femininity was tested only for the female targets, and relatedly, masculinity was tested for the male targets as a proxy for correct gender recognition. Whereas both femininity and masculinity can be present in any person to different degrees, this typically has no impact on correctly identifying someone as a man or a woman (see, e.g., Schug, Alt, & Klauer, 2015, for a gendered race effect). Furthermore, because ethnicity should not play a role, we also checked for the perceived German prototypicality of the women and men, *t*(96) = –1.89, *p* = .06, *d* = 0.38; both the men (*M* = 5.45, *SD* = 1.08) and women (*M* = 5.27, *SD* = 1.25) were perceived as highly prototypical of Germans.

All targets used in this experiment were matched to our participants on age and ethnicity; that is, the targets were all White (Germans) and in the 22–29 age range (*M*_*age*_ = 24.29, *SD* = 2.19) typical of a student population. They were presented in color against a black background in a portrait format. All of them wore a black T-shirt with only their shoulders visible. The same setting was used for both video clips (first part of the experiment) and for the individual pictures (second part of the experiment). Both the female and male targets had either short hair or hair gathered back in a ponytail in the back, for half of them (two men and two women) the face was completely free from hair, and the other half had hair drawn over one side of their forehead (without obstructing the face). No individual defining features (e.g., scars) were visible. Whereas gender could potentially be used to create shared group membership, this has not been the case in similar experiments looking at gender-based categorization (e.g., Klauer & Wegener, 1998), in which, as in our case, both the targets and the participants shared the same superordinate group (i.e., students). Also, the targets were introduced as students only at the beginning of the experiment, and gender was never explicitly mentioned.

In the experiment, we used a total of 96 statements on different university-related topics (e.g., library, exams, lecturers). These statements were adopted from Klauer and Wegener (1998) and have also been used in other WSW paradigm experiments (e.g., Rakić et al., 2011); the content of the statements was kept gender neutral so that it could not be used to aid categorization (e.g., “Exam dates should be given earlier in term”). Additionally, care was taken that each target made statements on different topics but that the topics were consistent across targets (e.g., each person spoke about the library, the seminar rooms, etc.). Given the large number of similar statements, we expected high error rates, allowing for the analysis of all response categories.

### Procedure

During the study phase, four German men and four German women were introduced as a group of university students and were observed as they made a total of 96 statements related to the university. Each of them made 12 different statements (e.g., “The library should have longer opening hours”), with each statement being presented in a video clip. All statements were presented in a random order. At the end of the discussion, participants were given the instruction for the test phase of the experiment.

According to the original WSW procedure, each statement is presented on a screen against the lineup of all eight targets and participants are required to press a number corresponding to a person they believe has said that statement earlier. To avoid too much noise for the ERP analyses, we simplified this procedure. During each trial, participants were asked to make a simple binary decision, yes/no, whether the person in the picture was the one who had made the statement (to avoid EEG artifacts due to eye movements). Each trial started with a fixation cross, presented for 1,000 ms. Then a sentence from the study phase was presented on the computer screen for 8,000 ms, followed by a picture of the face of one stimulus person for 3,000 ms. In 32 of the trials, this was the person who had said the sentence, implying that the correct response was *yes*; in another 32 trials, it was a wrong-person, same-gender distractor; and in the other 32, it was a wrong-person, different-gender distractor, implying that *no* responses were correct. Participants were asked to answer *yes* or *no*—that is, whether the person was correct or incorrect with regard to the statement—within a response window of 3,000 ms during the presentation of the face. This was done to avoid the influence of preparatory motor activity on the face-elicited ERPs. For each type of trial (e.g., same-gender distractor), the computer program registered one of three possible responses: *yes* response, *no* response, or not responded within time window.

A total of three blocks of trials were presented, with brief breaks between them. In each block, one-third of the statements were paired with the same person, one-third with a same-gender distractor, and one-third with a different-gender distractor; the difference between blocks was that statements paired with the same person in one block were paired with either a same-gender or a different-gender distractor in the other two blocks. Hence, the statements and picture pairs were rotated across blocks, but the order of the three blocks was counterbalanced and the statement–face pairs within each block were randomly presented. By doing so, we arrived at a sufficient number of trials to yield reliable ERPs, and we avoided any confounds from presenting each statement in all three types of trials.

#### Event-related potentials: Recording and analysis

EEG was recorded using a 64-channel BioSemi Active II system (BioSemi, Amsterdam, Netherlands). Active sintered Ag/AgCl electrodes were mounted in an elastic cap, and the EEG was recorded continuously with a 512-Hz sampling rate from DC to 155 Hz. BioSemi systems work with a “zero-ref” setup with ground and reference electrodes replaced by a CMS/DRL circuit (cf. www.biosemi.com/faq/cms&drl.htm). Blink artifacts were corrected using the algorithm implemented in BESA 5.3 (MEGIS Software GmbH, Graefelfing, Germany). The EEG was segmented relative to face onset from – 200 to 1,000 ms, with a 200-ms baseline. Trials contaminated by nonocular artifacts and saccades were rejected by using an amplitude threshold of 100 *μ*V and a gradient criterion of 75 *μ*V. Remaining trials were recalculated to the average reference, averaged relative to face stimulus onset separately for *yes* and *no* responses in the same-person, same-gender, and different-gender conditions, and digitally low-pass-filtered at 40 Hz (12 dB/octave, zero phase shift).

ERPs were analyzed at the left- and right-hemispheric temporal (TP9/TP10, P9/P10) and occipito-temporal electrodes (PO9/PO10). Mean amplitudes were calculated for the N170 (140–170 ms), as well as for the P2 (200–300 ms) and N250 (300–400 ms). Mean amplitude measures were statistically compared using repeated measures analyses of variance (ANOVAs). When appropriate, degrees of freedom were corrected according to the Greenhouse–Geisser procedure.

## Results

In all analyses in the present article, significance tests were conducted with *α* = .05.

### Behavioral data

#### Response omissions

There were fewer response omissions when photos of different-gender distractors were shown (*M* = .13, *SEM* = ±.03) than in the case of the same person or a same-gender distractor (*M*s = .15, *SEM*s = ±.03). This numerical difference was confirmed by a three-level (stimulus category: same person, same-gender distractor, different-gender distractor) analysis of variance (ANOVA) with repeated measures. There was a trend toward a main effect of stimulus category, *F*(2, 20) = 3.16, *p* = .064, Pillai’s trace = .24. Subsequent difference contrasts showed no difference between omissions after the same person or a same-gender distractor had been shown (*F* < 1), but these two stimulus categories led to more response omissions than did the different-gender distractor condition, *F*(1, 21) = 6.57, *p* = .02, *η*_p_^2^ = .24. This suggests that different-gender distractors were easier to process than the other two types of stimuli (confirming Hypothesis 1).

#### Reaction times

**Fig. 2:**
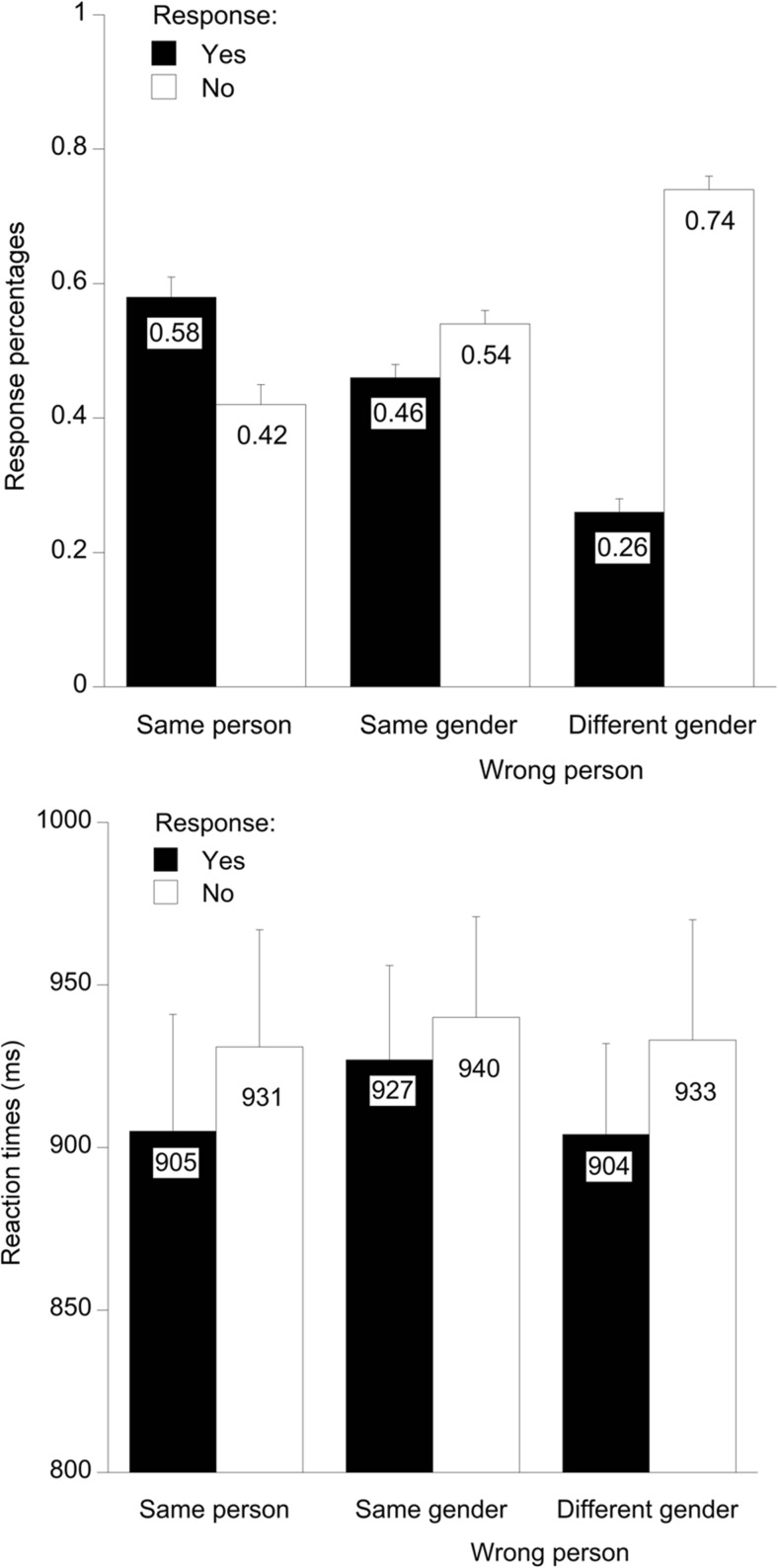
Mean proportions of *yes* and *no* responses (upper panel) and the associated reaction times (lower panel), separately for trials in which the same person, a same-gender distractor, or a different-gender distractor was shown. Error bars represent standard errors of the means

We next tested whether reaction times depended on stimulus category. The reaction times of *yes* and *no* responses pertaining to each stimulus category are shown in Fig. 2. There was a multivariate main effect of stimulus category, *F*(2, 20) = 3.84, *p* = .04, Pillai’s trace = .28. Within-subjects contrasts showed no linear trend of stimulus category (*F* < 1), but a quadratic trend showed that responses were slower for same-gender distractors than for the other two stimulus categories (*M*s = 933 vs. 918 ms), *F*(1, 21) = 7.23, *p* = .01, *η*_p_^2^ = .26. In other words, same-gender distractors were more difficult to react to than different-gender distractors (Hypothesis 2) and the same person.

#### Yes responses

To take into account differences in response omissions, we computed the proportions of *yes* responses and *no* responses among the total responses given (i.e., both *yes* and *no* responses; see Fig. 2). The same three-level ANOVA as above on *yes* responses yielded a main effect of stimulus category, *F*(2, 20) = 88.67, *p* < .001, *η*_p_^2^ = .90. Helmert contrasts showed more *yes* responses for the same person than for the two types of distractors, *F*(1, 21) = 105.34, *p* < .001, *η*_p_^2^ = .83. More interestingly, there were also more erroneous *yes* responses for same-gender than for different-gender distractors, *F*(1, 21) = 126.70, *p* < .001, *η*_p_^2^ = .86. Although the error levels were generally high, all proportions of *yes* and *no* responses shown in Fig. 2 are significantly different from chance level (.50), as shown by six one-sample *t* tests, all *t*s (21) > 167.33, all *p*s < .001. These findings corroborate Hypothesis 3 and are in line with the idea that same-gender distractors are more difficult to reject than different-gender distractors because of category memory.^1^

Taken together, the analyses of the behavioral data demonstrate that different-gender distractors were easier to respond to than the other categories, and that same-gender distractors were reacted to more slowly and were more often falsely accepted as correct than were different-gender distractors, demonstrating category memory.

### ERP results

A repeated measures ANOVA on N170 mean amplitudes (140–170 ms) with the within-subjects factors stimulus category (same person, same gender, different gender), response (*yes*, *no*), hemisphere (left, right), and site (TP, P, PO) yielded no significant effects, all *F*s < 3.20, all *p*s > .055, all *η*_p_^2^s < .15. Similarly, a corresponding ANOVA in the P2 time window (200–300 ms) did not result in any significant effects, all *F*s < 2.93, all *p*s > .076, all *η*_p_^2^s < .14.

**Fig. 3:**
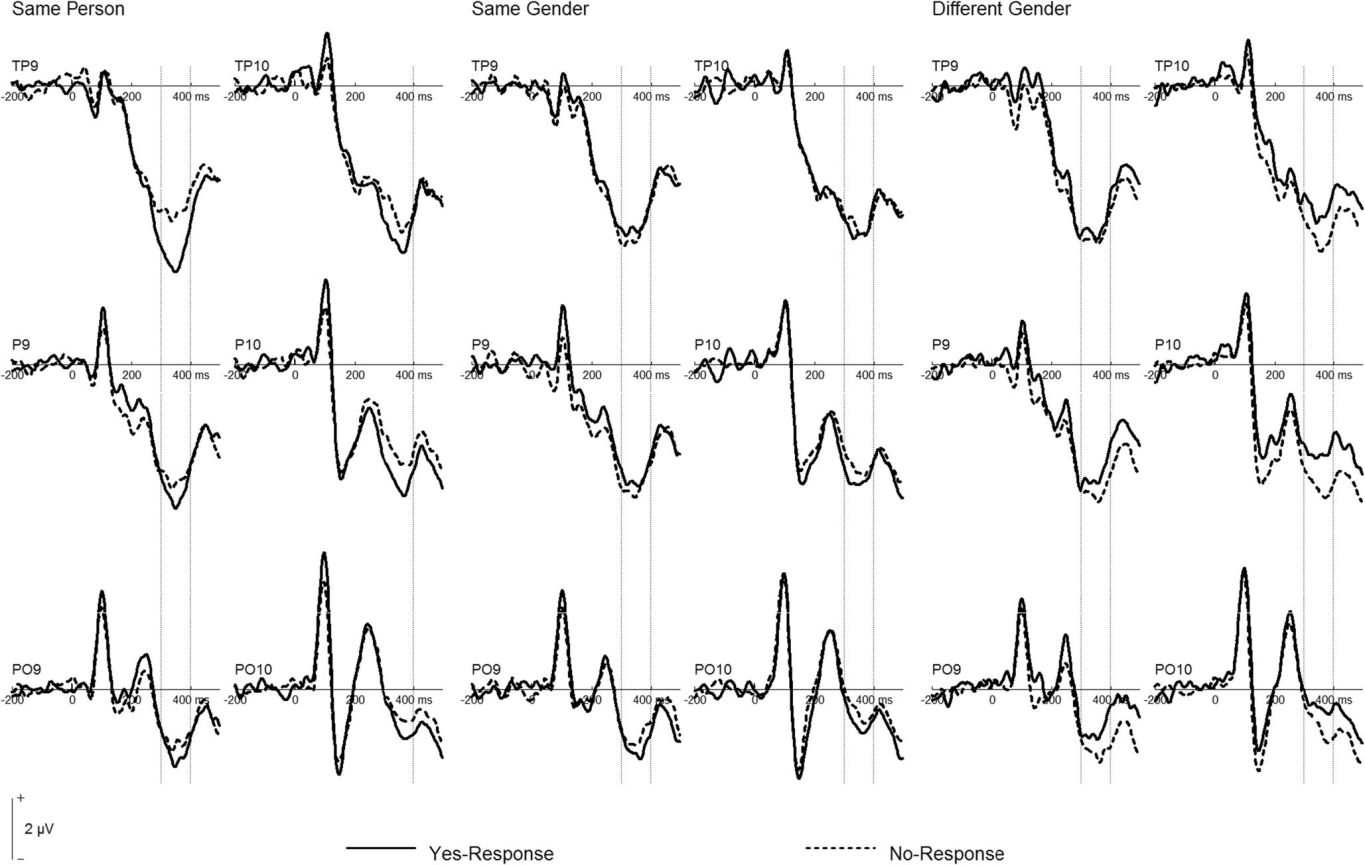
Grand mean event-related potentials at left and right temporal (TP9/TP10, P9/P10) and occipito-temporal (PO9/PO10) electrodes. Dashed vertical lines mark the N250 time range (300–400 ms). Note that positive is plotted upward

An ANOVA in the N250 time window (300–400 ms) revealed a significant Stimulus Category × Response interaction, *F*(2, 38) = 7.68, *p* = .002, *η*_p_^2^ = .29, which was further qualified by a Site × Stimulus Category × Response interaction, *F*(2, 38) = 3.55, *p* = .022, *η*_p_^2^ = .16. Post-hoc tests at electrodes TP9/TP10 (see Fig. 3) yielded significantly more negative amplitudes for *yes* than for *no* responses for the same person, *F*(1, 19) = 13.39, *p* = .002, *η*_p_^2^ = .41; no difference between *yes* and *no* responses for the same-gender condition, *F* < 1; and a trend toward more negative amplitudes for *no* than for *yes* responses in the different-gender condition, *F*(1, 19) = 3.95, *p* = .062, *η*_p_^2^ = .17. At electrodes P9/P10, a trend toward more negative amplitudes for *yes* responses was detected in the same-person condition, *F*(1, 19) = 3.07, *p* = .096, *η*_p_^2^ = .14, whereas no difference was observed for the same-gender condition, *F* < 1. Again, more negative amplitudes for *no* responses were detected in the different-gender condition, *F*(1, 19) = 10.28, *p* = .005, *η*_p_^2^ = .35. At electrodes PO9/PO10, neither the same-person, *F*(1, 19) = 1.52, *p* = .23, *η*_p_^2^ = .07, nor the same-gender condition yielded significant differences between *yes* and *no* responses, *F*(1, 19) = 1.05, *p* = .32, *η*_p_^2^ = .05. However, more negative amplitudes for *no* responses were detected in the different-gender condition, *F*(1, 19) = 5.68, *p* = .028, *η*_p_^2^ = .23. Accordingly, for both the same-person and different-gender conditions, the N250 was more negative for the correct response than for the incorrect response (Hypotheses 4 and 5), whereas no difference was observed for the same-gender condition.

**Fig. 4:**
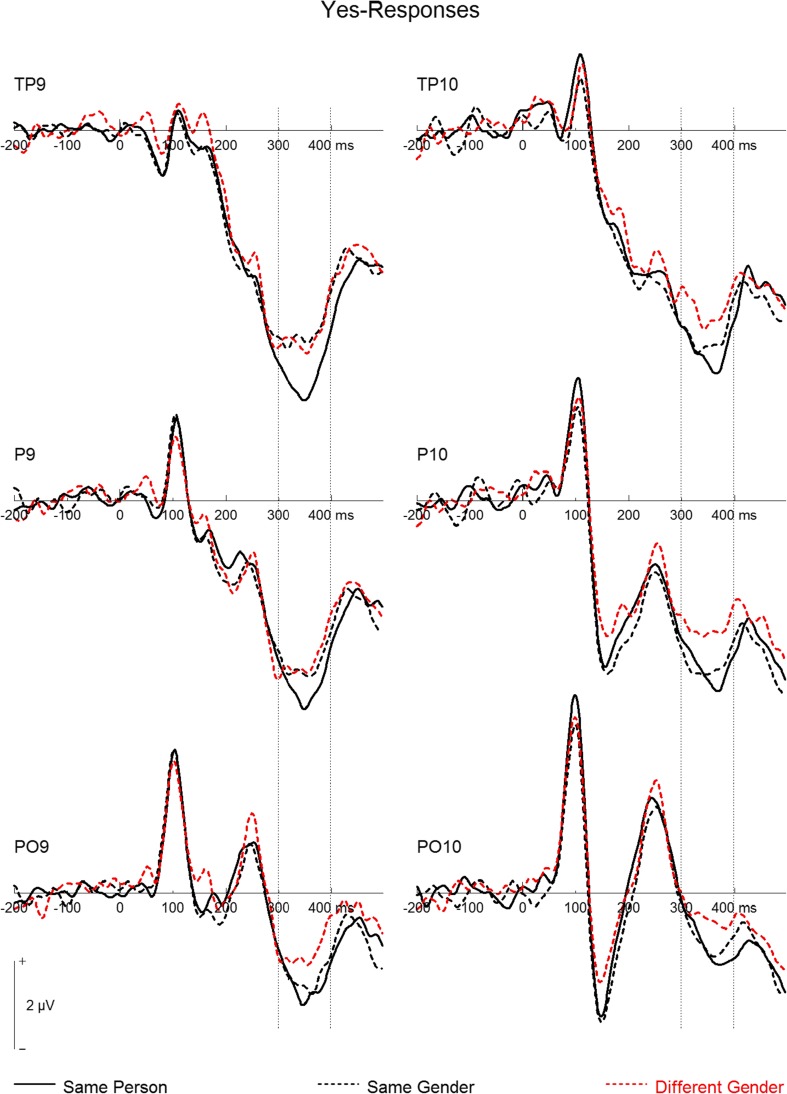
Grand mean event-related potentials for *yes* responses at left and right temporal (TP9/TP10, P9/P10) and occipito-temporal (PO9/PO10) electrodes. Dashed vertical lines mark the N250 time range (300–400 ms). Note that positive is plotted upward

Observing the interaction from a different angle, for *yes* responses (see Fig. 4) at TP sites, we observed a trend toward more negative amplitudes in the same-person relative to the same-gender condition, *F*(1, 19) = 3.76, *p* = .068, *η*_p_^2^ = .17. Whereas the same-person condition was significantly more negative than the different-gender condition, *F*(1, 19) = 8.71, *p* = .008, *η*_p_^2^ = .31, no difference between same gender and different gender was detected, *F*(1, 19) = 1.02, *p* = .325, *η*_p_^2^ = .05. At P sites, the same-person condition was not different from the same-gender condition, *F*(1, 19) = 0.753, *p* = .396, *η*_p_^2^ = .04, but it was more negative than the different-gender condition, *F*(1, 19) = 6.64, *p* = .018, *η*_p_^2^ = .26. Moreover, the same-gender condition was also more negative than the different-gender condition, *F*(1, 19) = 5.55, *p* = .029, *η*_p_^2^ = .23. Finally, at PO sites, *yes* responses were similar for the same-person and same-gender conditions, *F*(1, 19) = 0.19, *p* = .671, *η*_p_^2^ = .01, but elicited more negative amplitudes in the same-person than in the different-gender condition, *F*(1, 19) = 4.80, *p* = .041, *η*_p_^2^ = .20. A corresponding trend was observed for the comparison of the same-gender and different-gender conditions, *F*(1, 19) = 4.34, *p* = .051, *η*_p_^2^ = .19.

**Fig. 5:**
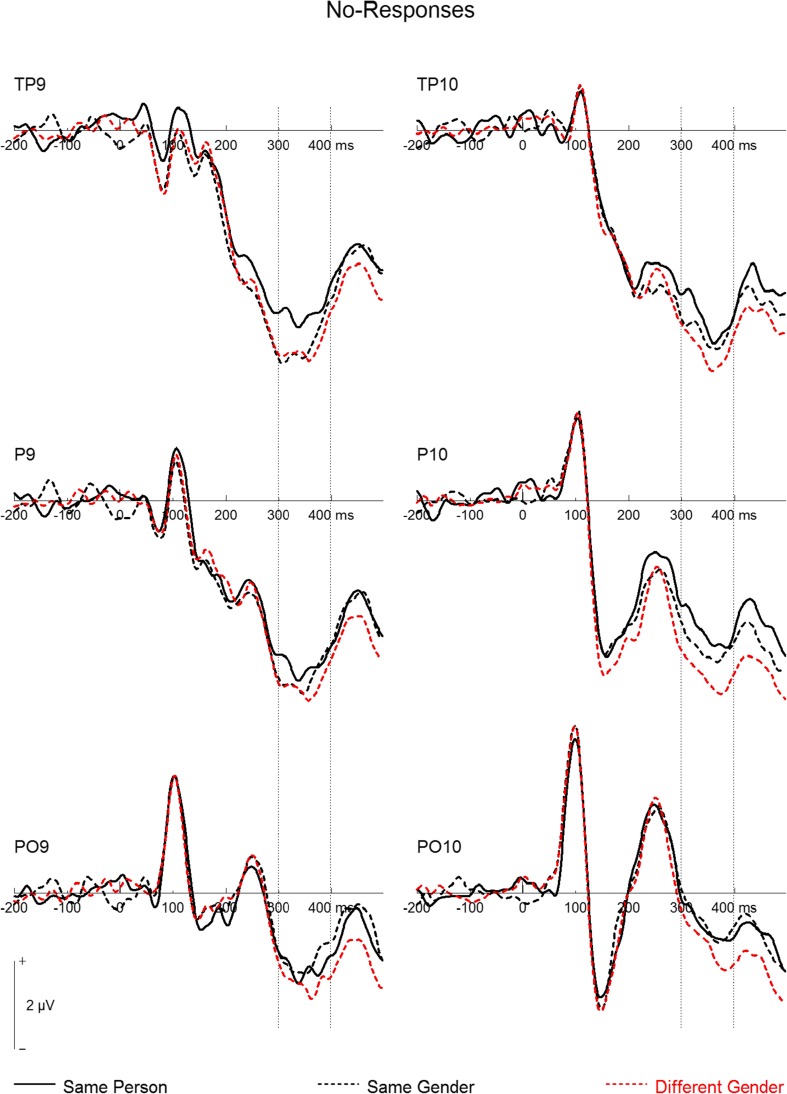
Grand mean event-related potentials for *no* responses at left and right temporal (TP9/TP10, P9/P10) and occipito-temporal (PO9/PO10) electrodes. Dashed vertical lines mark the N250 time range (300–400 ms). Note that positive is plotted upward

For *no* responses (see Fig. 5) at TP sites, we found a trend toward less negative amplitudes in the same-person than in the same-gender condition, *F*(1, 19) = 3.42, *p* = .080, *η*_p_^2^ = .15, and significantly less negative amplitudes in the same-person than in the different-gender condition, *F*(1, 19) = 8.13, *p* = .010, *η*_p_^2^ = .30. Again, the same-gender and different-gender conditions did not differ, *F*(1, 19) = 0.96, *p* = .339, *η*_p_^2^ = .05. At P sites, *no* responses yielded no significant difference between the same-person and same-gender conditions, *F*(1, 19) = 1.03, *p* = .324, *η*_p_^2^ = .05, whereas the different-gender condition was more negative than the same-person, *F*(1, 19) = 5.45, *p* = .031, *η*_p_^2^ = .22, and same-gender, *F*(1, 19) = 3.11, *p* = .094, *η*_p_^2^ = .14, conditions. Finally, at PO sites, *no* responses were again similar for the same-person and same-gender conditions, *F*(1, 19) = 0.12, *p* = .729, *η*_p_^2^ = .01. No difference was detected between the same-person and different-gender conditions, *F*(1, 19) = 1.49, *p* = .237, *η*_p_^2^ = .07, whereas a trend toward more negative amplitudes in the different-gender than in the same-gender condition was found, *F*(1, 19) = 3.17, *p* = .091, *η*_p_^2^ = .14.

## Discussion

After observing a “discussion” among men and women on a gender-neutral topic, we asked participants to indicate for each statement whether the subsequent face showed the person who had made it. Reaction time data indicated that different-gender distractors were easiest to respond to (Hypothesis 1), and that same-gender distractors were reacted to most slowly (Hypothesis 2) and were more often falsely accepted as correct than different-gender distractors (Hypothesis 3). Whereas analyses in the N170 and P2 time windows (200–300 ms) revealed no significant effects, in the N250 time window, in both the same-person and different-gender conditions, ERPs were more negative for correct responses (Hypothesis 4), which was not the case in the same-gender condition. For *yes* responses at anterior sites (TP9/TP10), the same-person condition differed from the same-gender and different-gender conditions, which were similar. At the same time, at more posterior electrodes (PO9/PO10), the same-person and same-gender conditions were indistinguishable and different from the different-gender condition. We discuss each of these findings in turn.

First, the behavioral data corroborate that our adaption of the WSW paradigm provoked category memory, so ERP findings could be analyzed. Conceptually replicating previous work (e.g., Klauer & Wegener, 1998), our findings show that participants encoded discussants’ gender in the absence of any respective instructions and when gender was irrelevant regarding the content of the discussion. How do the findings that analyses in the N170 and P2 time windows (200–300 ms) revealed no significant effects relate to previous findings? The first finding is generally in line with previous work, showing no effect of gender categorization in the N170 (Mouchetant-Rostaing & Giard, 2003; Mouchetant-Rostaing et al., 2000) and no automatic activation of gender category information in this component (Wiese, Schweinberger, & Neumann, 2008b), except when participants are explicitly attending to gender (Tomelleri & Castelli, 2012). We therefore conclude that facial gender categorization does not occur at this early perceptual processing stage. Interestingly, the N170 distinguishes between faces from different ethnic backgrounds (see Wiese, 2013, for an overview) and between young and older adult faces (e.g., Komes, Schweinberger, & Wiese, 2015; Wiese, Schweinberger, & Hansen, 2008a), suggesting that categorization according to ethnicity and age may well occur at this processing stage. Together, these findings suggest that gender categorization is perceptually more difficult and/or cognitively more demanding and therefore requires more in-depth processing performed at later stages.

At the same time, our ERP results are somewhat harder to integrate with previous findings of explicit and implicit gender categorization in the P2 time range (Mouchetant-Rostaing & Giard, 2003; Mouchetant-Rostaing et al., 2000). A critical difference between the present and these previous studies is related to task set. In contrast to those studies, our participants were focusing on information about individual identity. In this case, processing of gender information may be delayed and occur in time ranges typically associated with identity processing and explicit memory (see below).

Critically, our ERP results in the N250 suggest processing of both individual identity and gender category information within the same time window. A first piece of evidence for this is that, in both the same-person and different-gender conditions, ERPs were more negative for correct responses (*yes* for same person, *no* for different-gender distractor). It thus seems that a neural response between 300 and 400 ms after stimulus onset differentiated between correct and incorrect faces in these two conditions. At the same time, no differential ERP response depending on correct versus incorrect responses was detected for the same-gender condition, indicating that a rejection of an incorrect same-gender distractor was not possible on the basis of this signal. The neural response in this time window may therefore reflect higher uncertainty and may contribute to the higher error rates observed in the same-gender condition.

As for *yes* responses at anterior sites (TP9/TP10), the same-person condition differed from the same-gender and different-gender conditions, which were similar. It thus appears that in case of a strong association between a sentence and the correct face (and the corresponding correct *yes* response), the representation of this face was preactivated when the sentence was presented. This preactivation led to a pronounced negativity in the N250 time range. In case of no or only a weak association between a particular sentence and the correct face (and the corresponding incorrect *no* response), a substantially reduced preactivation of the correct face only resulted in a small N250 response. It thus appears that the correct face elicited either a stronger or weaker response than incorrect faces of both genders, depending on whether or not participants were able to correctly remember it. This pattern of responses therefore seems to reflect the particularly strong or weak activation of individual face representations, which is generally in line with studies suggesting the N250 and N250r as neural markers of individual face identity (Gosling & Eimer, 2011; Kaufmann et al., 2009; Schweinberger & Burton, 2003).

At the same time, as we mentioned, at more posterior electrodes (PO9/PO10), the same-person and same-gender conditions were indistinguishable and different from the different-gender condition. Accordingly, neural processes at these more posterior sites distinguished between the correct versus incorrect gender category, but not between correct versus incorrect individuals within a given category. We suggest that the presentation of a particular sentence did not only activate an individual face representation, but also a representation of the gender category (male/female). In case of a strong link between a sentence and the correct category (*yes* responses in the same-person and same-gender conditions), ERPs were more negative relative to the incorrect category. In case of no or only a weak link between a sentence and the correct category (*no* responses in the same-person and same-gender conditions), the correct category elicited less negativity than the alternative category, presumably contributing to the subsequent incorrect response.

These findings therefore reflect two separate processes in the N250 time range, which are similar in timing but different with respect to their topography. The first is observed at more anterior electrode positions (i.e., TP9/TP10) and distinguishes between correct and incorrect individual faces. The second process observed at more posterior positions differentiates between correct and incorrect gender category information.

A potential limitation of this study is that behavioral data do not clearly discriminate between memory and guessing of statements. However, the combination of behavioral data and ERPs offers some insight: The statements that had a strong link with a correct category were more likely to lead to correct yes/no answers, and the related ERPs were more negative. More importantly, these findings show that when gender information and identity information are encoded and stored together with a neutral sentence, they are also automatically activated the next time the given sentence is presented. This could potentially have an impact on the evaluation of targets if there is a discrepancy between the category (e.g., gender) and the statement (e.g., a woman using stereotypical male expressions in speech), since there is already evidence that ERPs are sensitive to ethnic incongruence between appearance and accents (Hansen, Steffens, Rakić, & Wiese, 2017). Future studies could test what effect this can have on impression formation and the evaluation of people on the basis of whether associated information is perceived as belonging to them or not.

In conclusion, this experiment shows that even when assessing identifying information is required, an individual’s gender category is simultaneously activated. These identity and category activations were paralleled by temporally similar but topographically distinct neural responses, in accordance with distinct processes for identity and gender processing, as in classic face perception models (e.g., Bruce & Young, 1986). Taken together, these findings provide further evidence for the often-found automaticity of gender-based categorization, which is activated at the same time as identity information and, more importantly, is encoded even when observing a conversation in which gender is irrelevant.

### Author note

We thank Bettina Kamchen for help with the data collection. The present research was funded by grants of the German Research Foundation (DFG) to M.C.S. and T.R. (STE 938/10-2; FOR 1097) and to H.W. (WI 3219/4-1; FOR 1097). Data used in the current article is available under license: CC BY-NC at 10.17635/lancaster/researchdata/169.

## Footnotes

1 Because there were more women than men in our sample, we checked whether the women showed a preference for the female targets (due to shared group membership). We found that, overall, participants were more likely to say *yes* to a correct female target (*M* = 25.50, *SD* = 6.42) than to a correct male target (*M* = 22.45, *SD* = 8.15), *F*(1, 20) = 9.03, *p* = .007, *η*_p_^2^ = .31. However, the interaction with participants’ gender was not significant, *F*(1, 20) = 0.42, *p* = .523, *η*_p_^2^ = .02. Similarly, when analyzing the *yes* answers for the same-gender targets, we found that participants answered *yes* more often in the case of female (*M* = 20.14, *SD* = 5.91) than of male (*M* = 16.73, *SD* = 5.63) targets, *F*(1, 20) = 5.09, *p* = .035, *η*_p_^2^ = .20. However, again there was no difference in terms of participants’ gender, *F*(1, 20) = 0.21, *p* = .655, *η*_p_^2^ = .01. These results indicate that female targets were more salient, but more importantly, this was consistent for both female and male participants. Additionally, this is in line with previous findings indicating that both men and women show better memory for female than for male faces (except for gay men) (Steffens, Landmann, & Mecklenbräuker, 2013).
